# Supplementary material for: Changes in the mRNA expression of glycolysis-related enzymes of Candida albicans during inhibition of intramitochondrial catabolism under anaerobic condition
Source: PLoS One. 2023 Apr 19;18(4):e0284353. doi: 10.1371/journal.pone.0284353 (PMC10115278; doi:10.1371/journal.pone.0284353)
Supplement: Attachment — Submitted filename: Plos Comments.pdf [file pone.0284353.s001.pdf]

Comments: Not Recommended.

This study does not have any novelty, as we already knows about almost all the paradigm during anaerobic or hypoxia conditions in which cells produces frequent energy by glycolysis or HMP shunt and not through by OXPHOS mechanism in eukaryotic system. The other part of this study is to check only m-RNA expression are not only promising option to correct prediction of protein or enzyme expression, so for that authors should need to focus on proteomics and metabolomics domain for the better elucidation in anaerobic conditions in Candida Species.
