# Supplementary material for: Changes in the mRNA expression of glycolysis-related enzymes of Candida albicans during inhibition of intramitochondrial catabolism under anaerobic condition
Source: PLoS One. 2023 Apr 19;18(4):e0284353. doi: 10.1371/journal.pone.0284353 (PMC10115278; doi:10.1371/journal.pone.0284353)
Supplement: Attachment — Submitted filename: Reply_mail20230320.docx [file pone.0284353.s002.docx]

**March 20, 2023**

Dr. Atul Vashist

Academic Editor

*PLOS ONE*

Dear Dr. Vashist,

Thank you for your comments and suggestions for the improvement of our manuscript (PONE-D-22-29062) titled “**Changes in the mRNA expression of glycolysis-related enzymes of Candida albicans during inhibition of intramitochondrial catabolism under anaerobic condition**”.

We have incorporated these suggestions in the revised manuscript (revised text is indicated in red for your convenience).

We have also provided point-by-point responses to all comments raised.

We hope that our manuscript is now suitable for publication in your esteemed journal.

Should you have any further comments or questions, please contact us.

Thank you for your consideration and we look forward to hearing from you.

Sincerely,

Takanori Narita

Department of Veterinary Medicine, College of Bioresource Sciences, Nihon University, 1866 Kameino, Fujisawa, Kanagawa, 252-0880, Japan

Tel: +81 466843848; Fax: +81 466843848

Email: [narita.takanori@nihon-u.ac.jp](mailto:narita.takanori@nihon-u.ac.jp)

Reviewer #1: The manuscript is interesting, since it provides studies on the regulation of PFK1 as a regulator of glycolysis and possibly growth and physiology of C. albicans. As the authors propose, more studies are required, particularly the measurement of the actual activities of the different enzymes, as well as the levels of ATP under the different conditions used.

However, the experiments are a good start to go more deeply into the reagulation of metabolism, but more importanly, the infectivity of this yeast. Pehaps also, a comparative study with. *S. cerevisiae*, as a model yeast. should be included in future experiments.

**Thank you for your comment and suggestion. In our upcoming study, we intend to analyze other enzyme activities related to glucose metabolism and elucidate the pathogenicity of *C. albicans* in comparison with *S. cerevisiae* as a non-pathogen.**

Reviewer #2: This study does not have any novelty, as we already knows about almost all the paradigm during anaerobic or hypoxia conditions in which cells produces frequent energy by glycolysis or HMP shunt and not through by OXPHOS mechanism in eukaryotic system. The other part of this study is to check only m-RNA expression are not only promising option to correct prediction of protein or enzyme expression, so for that authors should need to focus on proteomics and metabolomics domain for the better elucidation in anaerobic conditions in Candida Species.

**There are limited reports that have focused on the alcoholic fermentation of the pathogen *C. albicans*, though there are more reports on the important model organism *S. cerevisiae*. This study focused on the ability of alcoholic fermentation of *C. albicans* to induce auto-brewery syndrome. Our results indicate that the alcohol production of *C. albicans* is involved in pathogenicity. We intend to investigate alcohol production by *C. albicans* by incorporating proteomics and metabolomics. In the revised manuscript, we discussed the relationship between pathogenicity and alcohol production by *C. albicans*.**

Reviewer #3: Candida albicans is a major opportunistic pathogen of human capable of causing both superficial and systemic infections. Systemic C. albicans infection has emerged as a major cause of morbidity and mortality in immunocompromised patients. In the present article, the authors have studied the differential mRNA expression of enzymes in the glycolysis and alcohol fermentation to prove that C. albicans obtains energy by carbohydrate catabolism in the early phase of environmental change that helps them survive in various parts of the host. However, the claims made in the article about the pathogenesis potential of C. albicans and glycolysis require better validation.

The following are my comments

Major comments

1. The introduction and discussion should be revised and strengthened

**Thank you for your suggestion. The relationship between pathogenicity and alcohol fermentation by *C. albicans* was further discussed in the revised Introduction and Discussion**

2. Discussion: There are no experiments to prove that auto brewery syndrome is due to the ability of C. albicans to rapidly respond to environmental changes. Further it cannot be claimed by the results of comparing just two C. albicans.

**We have revised the Discussion accordingly.**

3. The difference in gene expression among strains of candida in similar culture conditions needs to explained better with substantial experimental evidence.

**We have added text on the comparison of gene expression between the two strains in the revised Results “Quantitative reverse transcriptase PCR”.**

Minor comments

1. Introduction: Line 45-46 should be rephrased

**We have revised the text accordingly.**

2. Introduction: Line 67-68 should be rechecked. Regulation of PFK1 was studied after partial purification of the enzyme.

**We have revised the text accordingly.**

3. Methods: Line 81-84. Mention the read out of the growth curve assay. Was the number of yeast cell counted using a hemocytometer or by determining the OD or CFU?

**We used a hemocytometer to count the number of yeast cells. We have indicated that in the revised manuscript.**
